# Statin therapy: a potential adjuvant to immunotherapies in hepatocellular carcinoma

**DOI:** 10.3389/fphar.2024.1324140

**Published:** 2024-02-01

**Authors:** Jiao Wang, Chengyu Liu, Ronghua Hu, Licheng Wu, Chuanzhou Li

**Affiliations:** ^1^ Department of Laboratory Medicine, Wuhan Hospital of Traditional Chinese and Western Medicine, Tongji Medical College, Huazhong University of Science and Technology, Wuhan, China; ^2^ Department of Transfusion Medicine, Wuhan Hospital of Traditional Chinese and Western Medicine, Tongji Medical College, Huazhong University of Science and Technology, Wuhan, China; ^3^ School of Clinical Medicine, Nanchang Medical College, Nanchang, China; ^4^ Department of Medical Genetics, School of Basic Medicine, Tongji Medical College, Huazhong University of Science and Technology, Wuhan, China

**Keywords:** HCC, immunotherapy, statins, cholesterol, ICI, inflammation

## Abstract

Hepatocellular carcinoma (HCC) is one of the most prevalent cancers worldwide and accounts for more than 90% of primary liver cancer. The advent of immune checkpoint inhibitor (ICI)-related therapies combined with angiogenesis inhibition has revolutionized the treatment of HCC in late-stage and unresectable HCC, as ICIs alone were disappointing in treating HCC. In addition to the altered immune microenvironment, abnormal lipid metabolism in the liver has been extensively characterized in various types of HCC. Stains are known for their cholesterol-lowering properties and their long history of treating hypercholesterolemia and reducing cardiovascular disease risk. Apart from ICI and other conventional therapies, statins are frequently used by advanced HCC patients with dyslipidemia, which is often marked by the abnormal accumulation of cholesterol and fatty acids in the liver. Supported by a body of preclinical and clinical studies, statins may unexpectedly enhance the efficacy of ICI therapy in HCC patients through the regulation of inflammatory responses and the immune microenvironment. This review discusses the abnormal changes in lipid metabolism in HCC, summarizes the clinical evidence and benefits of stain use in HCC, and prospects the possible mechanistic actions of statins in transforming the immune microenvironment in HCC when combined with immunotherapies. Consequently, the use of statin therapy may emerge as a novel and valuable adjuvant for immunotherapies in HCC.

## 1 Introduction

Hepatocellular carcinoma (HCC) accounts for 90% of primary liver cancer, and its incidence, along with associated morbidity and mortality, is increasing globally (Podlasek et al., 2023). The majority of HCCs are secondary to chronic Hepatitis B or C infections (Coffin and He, 2023), which mostly lead to cirrhosis, a chronic stage of liver lesion before HCC where scar tissue replaces liver cells (Omar et al., 2023). Accumulating findings indicate that cellular and acellular components in the tumor microenvironment (TME) can reprogram tumor initiation, growth, invasion, metastasis, and response to therapies (Jin and Jin, 2020), through interactions among various cell types, including the tumor cells, immune cells, stromal cells, and blood vessels (Sangro et al., 2021; Llovet et al., 2022; Hosonuma and Yoshimura, 2023). In recent years, a more comprehensive understanding of the TME has facilitated the development of immunotherapies for various cancers (Hosonuma and Yoshimura, 2023). Compared to conventional therapies such as chemotherapy and radiation therapy, immunotherapies have demonstrated striking efficacy and much less adverse effects (Kamrani et al., 2023). Major immunotherapy strategies include immune checkpoint blockers or inhibitors (ICB/ICIs) and adoptive cell transfer (ACT), in addition to vaccines and virotherapy, the clinical value of which has not yet been fully validated. Despite promising and often unprecedented response rates with ICI therapy, a substantial portion of patients fail to benefit from this therapy. Consequently, applications of combinational therapies or precision medicine, biomarker discoveries, and efforts in overcoming drug resistance and reducing adverse effects also emerge endlessly (Kamrani et al., 2023).

Key nutrients such as glucose and amino acids maintain metabolic homeostasis within immune cells and tumor cells present in the TME (Zou and Green, 2023). Metabolic reprogramming in the TME alters tumor immunity leading to changes in immunotherapeutic response observed in tumor-bearing mice and patients with cancer (Zou and Green, 2023). Recently, the importance of lipid accumulation has been particularly underscored in different immune cell subsets and tumor cells in the TME; for instance: tumor-associated macrophages (TAMs) show increased levels of the scavenger receptor CD36, leading to accumulated intracellular lipid and elevated fatty acid oxidation (FAO), which in return contributes to pro-tumor TAM polarization (Su et al., 2020). Similarly, high expression of fatty acid transporter protein 2 in tumor-associated neutrophils causes augmented uptake of arachidonic acid and synthesis of immunosuppressive molecule prostaglandin E2 (PGE2) (Veglia et al., 2019). In addition, cholesterol metabolism has also been involved in regulating CD8^+^ T cells in the TME. Suppression of acetyl-CoA acetyltransferase 1 (ACAT1) responsible for cholesterol esterification or cholesterol transporter proprotein convertase subtilisin/kexin type 9 (PCSK9) improves the anti-tumor performance of CD8^+^ T cells and potentiates ICB therapy (Yang et al., 2016; Liu et al., 2020).

Statins are widely used as cholesterol-lowering drugs in clinical practice and were originally discovered as potent inhibitors of the rate-limiting enzyme 3-hydroxy-3-methylglutaryl coenzyme A (HMG-CoA) reductase (HMGCR) in the mevalonate pathway (Lee et al., 2014). Besides the canonical effects on cholesterol levels, statins show pleiotropic effects on various cellular activities, such as proliferation, cell adhesion and migration, immune regulation, and endothelial functions (Pedersen, 2010). Concerning the synthesis of sterols and isoprenoids resulting from the mevalonate pathway are shown to be crucial for tumor growth, statins are proven to reduce the risk of HCC in plenty of meta-analyses and observational studies (Zhong et al., 2016; Wong et al., 2021). A recent meta-analysis including 24 studies demonstrated a 46% decrease in HCC risk among statin users, which indicate the high potential effects of statins as an alternative option in chemoprophylaxis of high-risk HCC population (Islam et al., 2020). The underlying mechanisms of the protective effects of statins may involve inhibition of oncogene MYC, protein kinase B (AKT), and NF-κB pathways by statins, leading to the enhanced cytokine production of interleukin-6 (IL-6), tumor necrosis factor-α (TNF−α), and transforming growth factor-β1(TGF-β1) (Li et al., 2020a). Therefore, modulation of the tumor immune microenvironment (TIME) through cytokines or chemokines by statins now has been extensively recognized. Statins inhibit chemokine (C-C motif) ligand 3 (CCL3) secretion by primary lung cancer cells and suppress IL-6 and CCL2 production by mesenchymal stromal cells (MSCs), and disrupt the communication between lung cancer cells and MSCs (Galland et al., 2020). As a result, statins negatively affect the proliferation of primary lung cancer cells, inspiring the reuse of statins in targeting the TIME (Galland et al., 2020). In addition, statin therapy has surprisingly increased the sensitivity and function of natural killer (NK) cells, enhancing the spontaneous killing of tumor cells in colon cancer and melanoma (Janakiram et al., 2016; Zhu et al., 2021). More recently, immune-based therapeutic mechanisms of simvastatin in HCC are offering broad opportunities for its applications in HCC patients (Yu et al., 2022). These findings together have highlighted the beneficial role of statins in cancer therapy.

Most of cancers progress from the chronic diseases or symptoms, and in this context, initially prescribed drugs against the chronic disease such as statin and metformin might be continually administrated during cancer treatment. Emerging studies have reported the impacts of adjunct use of commonly prescribed drugs on the outcome of ICI therapy (Vos et al., 2022). For example, in a multicenter observational retrospective study, statins were being used by 19.4% of the patients with various types of cancer following PD/PD-L1 therapy, and the baseline use of statins was independently related to an increased objective response rate (ORS), but not with progression-free survival (PFS) and overall survival (OS) (Cortellini et al., 2020). In two cohorts of patients with non-small-cell lung cancer (NSCLC) and malignant pleural mesothelioma, the concomitant use of statins was significantly related to improved OS and PFS in patients received ICIs (Zhang et al., 2021). Similarly, a recent study also showed improved OS with statin use in PD/PD-L1 inhibitor-treated patients with NSCLC (Singh et al., 2022). To date, these conclusions have mostly been drawn from retrospective studies and meta-analyses, and prospective studies are warranted to validate these findings. In this review, we introduce advances in immune microenvironment-driven immunotherapies for HCC, describe varieties of aberrant lipid metabolism in the liver that guide the application of statin as a cholesterol-lowering agent in HCC treatment, and discuss the potential use of stain in future immune therapy strategies for combinational treatment, based on observations mostly from long-term population studies and clinical trials.

## 2 Lipid metabolism in HCC

### 2.1 Tumor microenvironment in HCC

The complexity and homeostasis in TME during the tumor occurrence and development have now been well recognized, and cancer cells evolve to escape from immune surveillance by establishing a TME, which manifests remarkable immune suppression and promotion on tumor progression and metastasis. The architecture of a characteristic TME consists of the extracellular matrix (ECM) elements, fibroblasts, myofibroblasts, adipose cells, immune and inflammatory cells, endothelial cells, and pericytes, supplemented with secreted cytokines, chemokines and enzymes (Yang et al., 2011; Chen et al., 2015a). In a such milieu, interactive crosstalk among these components together determines how the tumor mass grows (Yang et al., 2011). The recent advent of ICI therapy for cancer has revolutionized the treatment of HCC and led to new therapeutic standards, as well as a more profound understanding of tumorigenesis, indicating that cancer treatment should not only target cancer cells but also the surrounding TME. It is noted that all components in TME are not independently existed, and their intrinsic interconnections are usually way more complex than described. Many reviews have also comprehensively discussed the architecture of TME in HCC (Sangro et al., 2021; Llovet et al., 2022; Sas et al., 2022). In particular, recent studies using single-cell profiling and multiomic techniques have greatly advanced our knowledge of the ecosystem in primary, metastatic, and early-relapse HCC, as well as how immune system and tumor cells respond to HCC status, immune evasion, and immunotherapies (Zheng et al., 2017; Sun et al., 2021; Lu et al., 2022; Liu et al., 2023; Murai et al., 2023).

#### 2.1.1 Cellular components in TME of HCC

**FIGURE 1:**
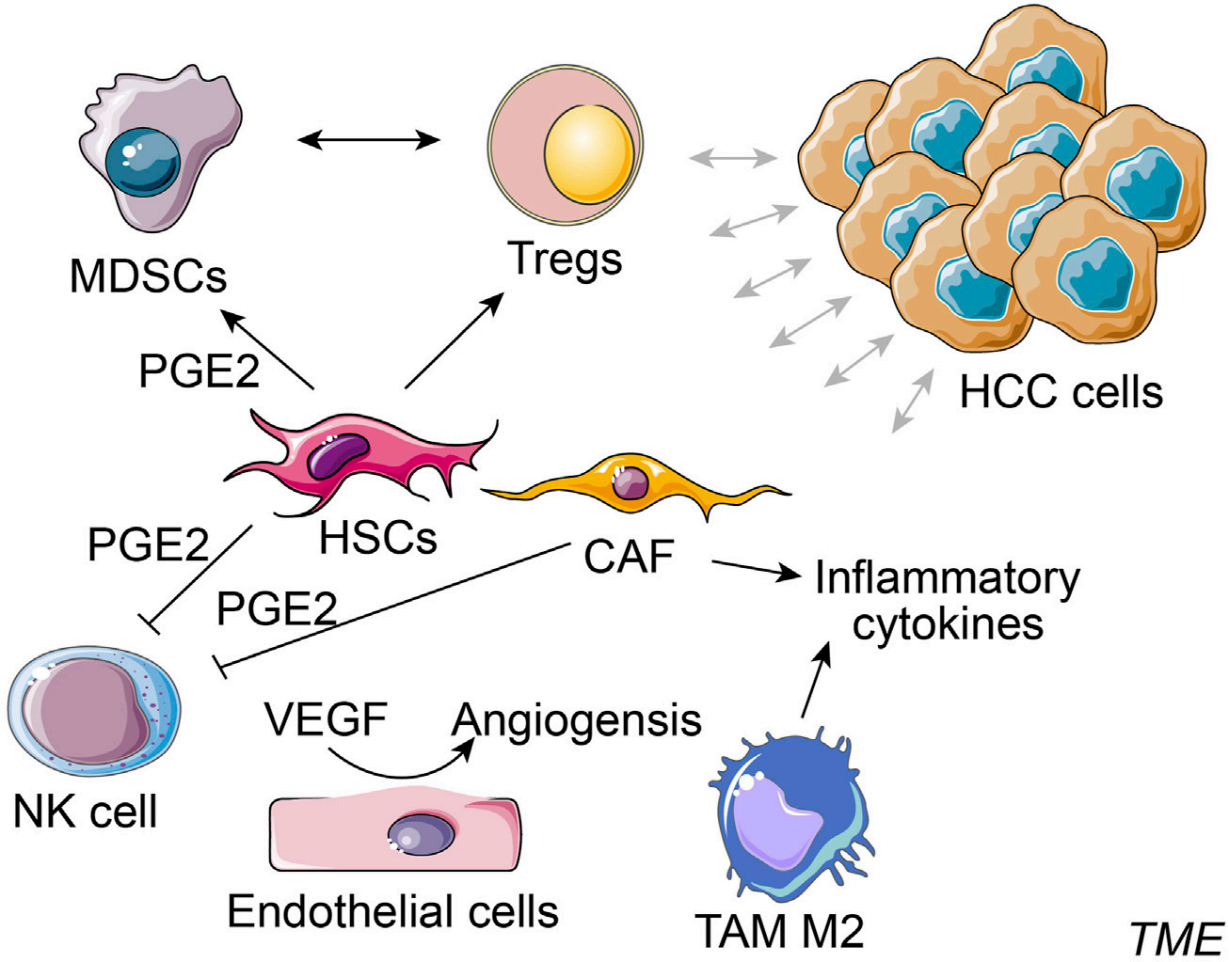
Simplified cellular components in TME of HCC. Typical cellular components in the tumor microenvironment (TME) of HCC consist of hepatocellular carcinoma (HCC) cells, hepatic stellate cells (HSCs), cancer-associated fibroblasts (CAFs), NK cell, regulatory T cells (Tregs), myeloid-derived suppressor cell (MDSCs), endothelial cells and tumor-associated macrophages (TAMs) M2, etc.

Typical cellular components in TME of HCC consist of hepatic stellate cells (HSCs), cancer-associated fibroblasts (CAFs), endothelial cells, NKs, regulatory T cells (Tregs), myeloid-derived suppressor cell (MDSCs) and TAMs (Figure 1) (Tahmasebi Birgani and Carloni, 2017). HSCs are a significant component in HCC-TME and are pivotal mediators of immunosuppression and pathogenesis of cirrhosis and HCC. HSCs provide an immunosuppressive niche for HCC by promoting the infiltration of Tregs and MDSCs (Zhao et al., 2014). MDSCs are important in immune suppression induced by inflammatory cytokines (e.g., PGE2), and MDSCs could affect the induction and function of Tregs. The inhibition of HSCs-derived PGE2 suppresses MDSCs accumulation induced by HSCs and HCC growth (Xu et al., 2016). CAFs secrete a higher amount of hepatocyte growth factor than the normal fibroblasts, and the secretion capacity of CAFs around HCC is positively correlated with the tumor size (Jia et al., 2013). CAFs-secreted C-C motif chemokine ligand proteins, CCL2, CCL5, CCL7, and CXCL16, promote the migration and invasion of HCC cells and enhance their metastasis to other organs by activation of the TGF signaling pathway (Liu et al., 2016). Alternatively, CAFs in TME also secrete PGE2 to suppress the NK cell function in HCC (Li et al., 2012). Tumor endothelial cells promote tumor angiogenesis and regulate cytotoxic T cells in the TME (Sakano et al., 2022; Feng et al., 2023b). In HCC, tumor endothelial cells could induce tumor-infiltrating T-cell exhaustion and induce the suppression of tumor growth via silencing glycoprotein non-metastatic melanoma protein B expression (Sakano et al., 2022). Like other cancers, liver malignancies and HCC progression are also facilitated by TAMs at TME of HCC (Shirabe et al., 2012). A high abundance of M2 macrophage has been shown to correlate with aggressive phenotypes of HCC (Dong et al., 2016). These alternatively activated macrophages release high levels of pro-metastatic cytokines in the circulation of HCC patients, such as IL-6, IL-1, and TNF-α (Ataseven et al., 2006). TAMs, by activating the STAT3 signaling in HCC cells, are associated with large tumor size, intrahepatic metastasis, and a high rate of HCC recurrence (Peng et al., 2005; Mano et al., 2013). Taken together, the complex cellular components in TME of HCC dynamically interact through cell-cell contacts and cytokine signaling and exhibit considerable influences on tumor immune responses (Chen et al., 2023a).

#### 2.1.2 Non-cellular components in TME of HCC

ECM containing extracellular substances, tumor vasculature system, exosomes, and cytokines are major non-cellular components in TME of HCC (Tahmasebi Birgani and Carloni, 2017). Matrix metalloproteinases (MMPs) are remodeler enzymes responsible for the degradation and remodeling of the ECM. Increased expression of MMP-9 was detected around the tumor capsule in HCC and was strongly correlated with tumor size, capsule status, tumor stage, and HCC recurrence risk (Arii et al., 1996; Sun et al., 2005). An upregulated level of pro-inflammatory cytokine IL-6 was found in the serum of HCC patients (Xu et al., 2021), and the IL-6/STAT3 signaling pathway has been frequently shown to be constitutively activated in HCC patients, which is associated with poor prognosis (Li et al., 2023a), affecting activities of anti-apoptosis, angiogenesis, proliferation, invasion, metastasis, and drug resistance of HCC cells (Xu et al., 2021). IFN-γ produced by mucosa-associated invariant T (MAIT) cells was reduced in the peripheral blood and liver of HCC patients than in the controls (Huang et al., 2021). Moreover, increased vascular endothelial growth factor (VEGF) in the serum of HCC patients strongly dictates the severities of tumor invasiveness, metastasis, and poor prognosis of patients (Li et al., 1999; Poon et al., 2004; Lacin and Yalcin, 2020), while suppression of VEGF mitigates the angiogenesis and prevents the proliferation and growth of HCC cells (Raskopf et al., 2008).

### 2.2 Abnormal lipid metabolism (dyslipidemia) in HCC

As another critical target for cancer therapy, cancer metabolism is regulated by cell-intrinsic factors and metabolite availability in the TME. Key modulations are dependent on tumor cell metabolism, cell interactions in TME, tumor heterogeneity, and whole-body metabolism homeostasis (Elia and Haigis, 2021). Consequently, specific metabolic adaptations by TMEs drive further cancer progression. Aberrant lipid metabolism in HCC generally manifests as alternations in lipid uptake and efflux, dysregulated endogenous lipid synthesis, elevated cholesterol esterification, and disruptions in lipid oxidation. These alterations are intimately linked with tumor activities in HCC (Feng et al., 2023a).

#### 2.2.1 Lipid metabolism in the normal liver

The liver is the second largest organ in the body where a variety of metabolic activities take place. In this review, other than protein metabolism and glucose metabolism, we briefly discuss lipid metabolism in normal liver and HCC. Lipids in the body include triglycerides, phospholipids, and cholesterol, and the first two are composed of fatty acids. Triglycerides are mainly used as an energy store in case of high energy demand, whereas cholesterol and phospholipids are the source materials for the synthesis of the cell membrane and steroid hormones, respectively (Borén and Taskinen, 2022). Lipogenesis occurs in the fat cells and hepatocytes by converting excess acetyl CoA generated by glycolysis into fatty acids, triglycerides, cholesterol, steroids, and bile salts (Desoye and Herrera, 2021). When energy is needed from the fat stored in adipose tissue, the process of lipolysis initiates by hydrolyzing triglycerides into fatty acids and glycerol which further enter the circulation to be transported to tissues such as the liver. Then in the liver, glycerol enters the glycolysis pathway after converted into glycerol-3-phosphate and fatty acids undergo β-oxidation and enter the tricarboxylic acid cycle to release ATP (Feng et al., 2023a).

#### 2.2.2 Fatty acid metabolism in HCC

Increased uptake of extracellular fatty acids promotes epithelial-mesenchymal transition, cell growth, and proliferation in HCC by mechanisms that induction of CD36/fatty acid translocase is strongly engaged (Nath et al., 2015; Pascual et al., 2017). In line with this, the synthesis of fatty acids in HCC cells is atypically higher, due to the disrupted expression or activities of key enzymes involved during the synthesis process, including malonyl-CoA, acetyl-CoA, and fatty acid synthase (FASN). Overexpression of FASN was evidenced to promote the carcinogenesis of HCC (Wang et al., 2022). Inactivation of FASN impairs hepatocarcinogenesis driven by AKT and pharmacological blockade of FASN might be highly useful in the treatment of human HCC (Li et al., 2016). β-oxidation of fatty acids (FAO), a process of lipolysis taking place at the mitochondria, is found deficient in HCC (Fujiwara et al., 2018). Peroxisome-proliferator-activated receptors (PPARs) are transcription factors that are activated by endogenous fatty acids and fatty acid derivatives. PPARα is a major transcriptional regulator of fatty acid oxidation and extended PPARα activation causes HCC in rodent mice by mechanisms that involve perturbation of the cell cycle and production of ROS [reviewed by Michalik et al. (2004)]. Overall, the extracellular uptake, biosynthesis, and degradation of fatty acids are reinforced in HCC progression.

#### 2.2.3 Cholesterol metabolism in HCC

Given the low expression of low-density lipoprotein receptor (LDLR), the transmembrane receptor for cholesterol, facilitates upregulated cholesterol synthesis, the expression of LDLR seems to be notably lower than that in normal cells surrounding the tumor in HCC (Chen et al., 2021). In addition to the LDLR-mediated cholesterol uptake, the efflux of cholesterol has also been shown to be downregulated in HCC (Cui et al., 2020). Upregulated cholesterol esterification is another indication of HCC-associated cholesterol metabolism. Cholesterols are stored as cholesterol esters after cholesterol esterification and provide a critical energy supply for tumor cells (Tosi and Tugnoli, 2005). Alternatively, cholesterol can be oxidized into oxysterols which later impact the TME by promoting immunosuppression and assist tumor metastasis (Xia et al., 2021).

## 3 Lipid-targeting statins in HCC

HMG-CoA inhibitors statins are a class of small bioactive molecules designed decades ago to reduce cholesterol levels and therefore are routinely used to tackle many cardiovascular diseases (CVD). Accumulating evidence has demonstrated the multifaceted impacts of statins on the metabolism of lipoproteins, such as chylomicrons and high-density lipoproteins (HDLs) (Lamon-Fava, 2013).

Notably, the development and application of statins have ushered in a new era in the prevention and treatment of CVDs such as coronary heart disease and hypertension. For the primary and secondary prevention of coronary heart disease, statins have been identified as the first choice for hypercholesterolemia. Statins include lovastatin (Altoprev), pravastatin, simvastatin (Zocor), fluvastatin (Lescol XL), atorvastatin (Lipitor), cerivastatin, bervastatin, niavastatin, pitavastatin (Livalo) and rosuvastatin (Crestor), etc. The first six statins have been approved by the Food and Drug Administration (FDA), and the benefits and advantages of statins have now been extensively recognized (Pedersen, 2010; Adhyaru and Jacobson, 2018).

### 3.1 Anti-tumor effects of statins in HCC

**FIGURE 2:**
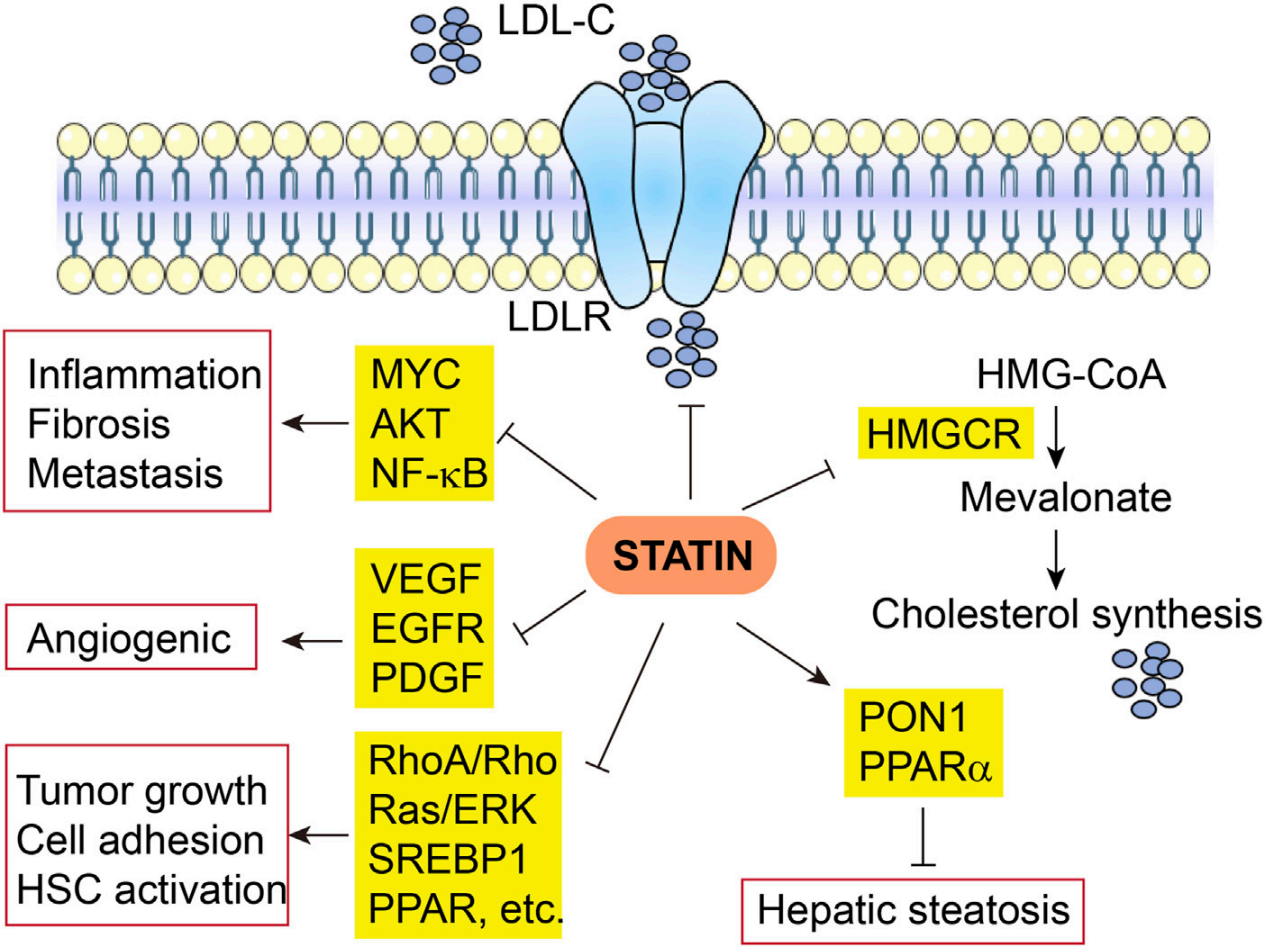
Multifaceted mechanisms of actin of statin in HCC. LDL-C, low-density lipoprotein cholesterol; LDLR, low-density lipoprotein receptor; HMGCR, 3-Hydroxy-3-Methylglutaryl-CoA reductase.

A promising role of statins in the prevention and relapse protection of HCC has been suggested by several retrospective observational trials showing the efficacy of statins in reducing the risk and recurrence of HCC and other cancers (Goh and Sinn, 2022) (Table 1). The mechanisms of action are broadly categorized below into inflammation and non-inflammation related functions (Figure 2).

**TABLE 1:** Representative observational studies regarding statins use in HCC and other cancers.

Study	Therapy	Cancer type	Study type	Patient number	Findings
Zeng et al. (2023)	Statins	HCC	Meta-analysis	1,774,476	Statin use was associated with reduced HCC risk (HR: 0.52; 95% CI, 0.37–0.72)
Khazaaleh et al. (2022)	Statins	HCC	Meta-analysis	2,668,497	Significant risk reduction of HCC among all statin users with a pooled OR of 0.573 (95% CI: 0.491–0.668, *p* < 0.05) compared to non-statin users
Vahedian-Azimi et al. (2021)	Statins	HCC	Meta-analysis	195,602	Statin use was associated with lower risk of mortality in people with HCC or cirrhosis, but it was not significant due to the large confidence interval [OR (95% CI) = 0.32 (0.09, 1.15), *p* = 0.210]
Islam et al. (2020)	Statins	HCC	Meta-analysis	59,703	Statin use was associated with a reduced risk of HCC development (risk ratio, 0.54; 95% CI, 0.47–0.61) compared with non-statin users, supporting the beneficial inhibitory effect of statins on HCC incidence
Li et al. (2020b)	Statins	HCC	Meta-analysis	62,273	Statin use was associated with a reduced all-cause mortality in HCC patients [risk ratio (RR): 0.81, 95% CI: 0.74–0.88, *p <* 0.001]
Santoni et al. (2022)	Statins	Kidney cancer	Retrospective cohort study	219	Statin use was associated with an apparently longer median OS (34.4 *versus* 18.6 months, *p* = 0.017) and PFS (11.7 *versus* 4.6 months, *p* = 0.013)
Nayan et al. (2017)	Statins	Kidney cancer	Meta-analysis	18,105	Statin use was not significantly associated with PFS (pooled HR 0.92, 95% CI, 0.51–1.65); however, statin use was associated with marked improvements in cancer-specific survival (pooled HR 0.67, 95% CI, 0.47–0.94) and overall survival (pooled HR 0.74, 95% CI, 0.63–0.88) in patients with kidney cancer
Allott et al. (2020)	Statins	Prostate cancer	Prospective cohort study	44,126	Current statin use was associated with lower risk of PTEN-null and lethal prostate cancer (HR, 0.40; 95% CI, 0.19–0.87; and HR, 0.76; 95% CI, 0.60–0.96; respectively)
Jespersen et al. (2014)	Statins	Prostate cancer	Case-control study	42,480	The use of statins was associated with a risk reduction of overall prostate cancer (adjusted OR, 0.94; 95% CI, 0.91–0.97) and specifically with advanced prostate cancer (adjusted OR, 0.90; 95% CI, 0.85–0.96)
Cardwell et al. (2015)	Statins (after cancer diagnosis)	Breast cancer	Retrospective cohort study	17,880	Statin use after a diagnosis of breast cancer reduced mortality due to breast cancer (adjusted HR, 0.84; 95% CI, 0.68–1.04)
Ahern et al. (2011)	Statins (simvastatin being the mostly prescribed lipophilic statin)	Breast cancer	Prospective cohort study	18,769	Simvastatin was associated with a reduced risk of breast cancer recurrence among Danish women diagnosed with stage I–III breast carcinoma (adjusted HR = 0.70, 95% CI, 0.57–0.86)
Takada et al. (2022)	Statins	Lung cancer	Propensity score-matched analysis	390	Statin use was associated with a significantly longer in the OS (*p* = 0.0433), but not the PFS (*p* = 0.2251) than those who did not receive statin therapy
Rossi et al. (2021)	Statins	Lung cancer	Retrospective cohort study	162	Statin use was associated with an apparently longer Median PFS (17.57 vs. 9.57 months, *p* = <0.001) and median OS was superior in the statin-users group, with a statistically significant difference (19.94 vs. 10.94 months, *p* = <0.001)
Ung et al. (2018)	Atorvastatin, simvastatin, lovastatin, pravastatin, and rosuvastatin (both pre- and post- cancer diagnosis)	Lung cancer	Retrospective cohort study	19,974	Overall baseline statin exposure was associated with a decrease in mortality risk for squamous-cell carcinoma patients (HR, 0.89; 95% CI, 0.82–0.96) and adenocarcinoma patients (HR, 0.87; 95% CI, 0.82–0.94), but not among those with SCLC. Post-diagnostic statin exposure was associated with prolonged survival in squamous-cell carcinoma patients (HR, 0.68; 95% CI, 0.59–0.79) and adenocarcinoma patients (HR, 0.78; 95% CI, 0.68–0.89). Baseline or post-diagnostic exposure to simvastatin and atorvastatin was associated with extended survival in NSCLC cancer subtypes
Cho et al. (2015)	Statins (before cancer diagnosis)	Non-Hodgkin lymphoma	Case-control study	18,657	Previous statin administration was associated with a reduced risk of subsequent non-Hodgkin lymphoma (adjusted OR, 0.52; 95% CI, 0.43–0.62)
Cote et al. (2019)	Statins (before cancer diagnosis)	Glioblastoma	Prospective cohort study	280,455	Ever statin use (HR, 1.43, 95% CI, 1.10–1.86) was significantly associated with increased glioma risk
Sperling et al. (2017)	Statins (before cancer diagnosis)	Endometrial cancer	Case-control study	77,509	The use of statins was not associated with the risk of endometrial cancer (OR, 1.03; 95% CI, 0.94–1.14). In addition, endometrial cancer risk did not vary substantially with duration or intensity of statin use
Li et al. (2021)	Statins	Colorectal cancer	Meta-analysis	387,518	The use of statins was significantly associated with a decrease in overall mortality (HR, 0.81; 95% CI, 0.76–0.86) and cancer-specific mortality (HR, 0.78; 95% CI, 0.72–0.85) of colorectal cancer
Cho et al. (2021)	Statins	Gastric cancer	Retrospective cohort study	80,271	Statin use was associated with a reduction of gastric cancer mortality in the general population but not with gastric cancer incidence
Nielsen et al. (2012)	Statins (before cancer diagnosis)	13 cancer types	Retrospective cohort study	295,925	Statin use in patients with cancer was associated with reduced cancer-related mortality. Multivariable-adjusted HR for statin users, as compared with patients who had never used statins, were 0.85 (95% CI, 0.83–0.87) for death from any cause and 0.85 (95% CI, 0.82–0.87) for death from cancer
Mei et al. (2017)	Statins	Miscellaneous	Meta-analysis	1,111,407	Statin use was significantly associated with decreased risk of all-cause mortality (HR, 0.70; 95% CI, 0.66–0.74) compared with non-statin users. The observed pooled estimates were retained for cancer-specific mortality (HR, 0.60; 95% CI, 0.47–0.77), PFS (HR, 0.67; 95% CI, 0.56–0.81), recurrence-free survival (HR, 0.74; 95% CI, 0.65–0.83) and disease-free survival (HR, 0.53; 95% Cl, 0.40–0.72)
Wang et al. (2016)	Statins	Miscellaneous	Prospective cohort study	146,326	In a cohort of postmenopausal women, regular use of statins or other lipid-lowering medications was associated with decreased cancer death (HR, 0.78; 95% CI, 0.71–0.86), regardless of the type, duration, or potency of statin medications used
Emberson et al. (2012)	Statins	Miscellaneous	Meta-analysis	175,000	A median of 5 years of statin therapy had no effect on the incidence of, or mortality from, any type of cancer (or the aggregate of all cancer)

Abbreviations: HCC, hepatocellular carcinoma; HR, hazard ratio; OR, odds ratio; OS, overall survival; PC, placebo-controlled; PFS, progression-free survival; RR, response rate.

#### 3.1.1 Inflammation related mechanisms

The anti-tumor effects of statins in HCC have been attributed to the inhibition of MYC oncogene (Cao et al., 2011), protein kinase B (AKT) (Roudier et al., 2006; Ghalali et al., 2017), and NF-κB pathways, as well as decreased production of pro-inflammatory cytokines (Wang et al., 2006; Li et al., 2020a). Fluvastatin blocked the activation and hepatic fibrogenesis of steatosis-induced HSCs by suppressing the generation of reactive oxygen species (ROS), NF-κB activity, and expression of pro-inflammatory genes (Chong et al., 2015). Rosuvastatin also decreases hepatic inflammation through downregulated expression of pro-inflammatory cytokines such as TNF-α, IL-6, and TGF-β1 and other tumor-associated growth factors (Yokohama et al., 2016). Nonalcoholic fatty liver disease (NAFLD) and non-alcoholic steatohepatitis (NASH) could be treated and prevented with statins owing to the diverse properties of statins via prevention of the liver from inflammation and fibrosis (Ahsan et al., 2020). Some animal studies indicate that statins significantly improve NASH-associated hepatic lipotoxicity, oxidative stress, inflammatory responses, and fibrosis (Park et al., 2016; Schierwagen et al., 2016; Ahsan et al., 2020). In NAFLD/NASH patients, the pleiotropic effects of statins are elaborated by decreased inflammation and fibrosis via modulation of cell proliferation, anti-oxidant, and anti-thrombotic activities (Chong et al., 2015).

#### 3.1.2 Non-inflammation related mechanisms

The anti-angiogenic effects of statins have been widely disclosed. Statins show a protective role against HCC as they decrease hepatic expression of angiogenic factors like VEGF receptor, epidermal growth factor receptor (EGFR), and platelet-derived growth factor (PDGF) (Dulak and Józkowicz, 2005; Yokohama et al., 2016). Simvastatin has been found to lessen tumor cell growth and impair tumor cell adhesion and invasion (Relja et al., 2011). Hepatic fibrosis is blocked by simvastatin via RhoA/Rho kinase and Ras/ERK pathways (Schierwagen et al., 2016). Atorvastatin use also reduces HSC activation via the production of sterol regulatory element-binding protein 1 (SREBP1) and peroxisomal proliferator-activated receptor (PPAR) (Marinho et al., 2017), in addition to the reduction in fibrosis and portal hypertension via non-canonical Hedgehog signaling (Uschner et al., 2015). In addition, statins also decrease isoprenoid biogenesis that is essential for cell survival (Schierwagen et al., 2016), resolve crown-like cholesterol crystals (Ioannou et al., 2015), and inactivate HSCs (Wang et al., 2013). In line with these findings, by downregulating the oxidative stress, statins decrease hepatic steatosis through increased hepatic antioxidant paraoxonase 1 (PON1) activity (Samy and Hassanian, 2011), and increase mitochondrial and peroxisomal oxidation, as well as expression of an FAO regulator PPAR-alpha (Park et al., 2016). Furthermore, statins ameliorate fibrogenesis in NASH through reestablishment of liver sinusoidal endothelial cell and HSCs phenotype and increasing endothelial nitric oxide synthase (eNOS) activity (Abraldes et al., 2007; Marrone et al., 2013; Rodríguez et al., 2017).

In summary, the pleiotropic anti-tumor effects of statin in HCC are not exclusively dependent on its impacts on HMGCR and downstream cholesterol biosynthesis, instead, via complex crosstalk, they interact frequently. For instance, cholesterol synthesis is not only directly downregulated by statin via the mevalonate pathway, but also modulated by SREBP1 and PPAR which are also suppressed by statin, in addition, NF-kB-induced inflammation can be ameliorated by direct effects of statin, as well indirect effects due to reduced cholesterol synthesis.

### 3.2 Clinical use of statins in HCC

#### 3.2.1 Monotherapy use of statins in HCC

Intriguingly but somehow disappointingly, indications from prospective interventional trials and studies remain inconclusive, although association of statin use with a decreased risk of HCC carcinogenesis and recurrence has been described (Table 2) (Chiu et al., 2011; Tsan et al., 2012; Butt et al., 2015; Kawaguchi et al., 2017; Khazaaleh et al., 2022). In virus-independent liver lesions, atorvastatin use (10 mg/day) in NASH patients indicated an improvement in liver functions by 74%, along with a rise in serum protein and lipid metabolism regulator adiponectin (Hyogo et al., 2008; Athyros et al., 2017). Sustained virologic response and lower risk of cirrhosis progression were consistently found among statin users (Butt et al., 2015). These evidences together indicate a general liver protective function of statin use.

**TABLE 2:** Representative intervention clinical studies regarding statins use in HCC.

Intervention	Cancer type	Trial ID	Study type	Patient number	Overall benefits	Findings
Pravastatin (40 mg/day) plus TAE and 5-FU	HCC	NR	Randomized trial	91	Positive	Pravastatin prolonged the survival of patients with advanced HCC (median survival, pravastatin group vs. controls, 18 months vs. 9 months, *p* = 0.006) (Kawata et al. (2001))
Pravastatin (20–40 mg/day) plus TACE	HCC	NR	Randomized trial	183	Positive	Pravastatin plus TACE prolonged the survival of patients with advanced HCC (median survival, pravastatin plus TACE group vs. TACE alone group, 20.9 months vs. 12.0 months, *p* = 0.003) (Graf et al. (2008))
Atorvastatin (A, 10 mg/day) and metformin (M) in combination (SAM) with Sorafenib (S)	HCC	CTRI/2018/07/014,865	Phase I, sequential cohorts	40	Positive regarding the adverse effects	The SAM combination in HCC patients with predominantly unfavorable baseline disease characteristics showed a marked reduction in sorafenib-related side effects. The median OS for patients without early hepatic decompensation (*n* = 31) was 8.9 months (95% CI: 3.2–14.5 months) (Ostwal et al. (2022))
Pravastatin (40 mg/day) plus sorafenib	HCC	NCT01075555	Phase III, randomized trial	312	Negative	Addition of pravastatin to sorafenib did not improve survival in patients with advanced HCC, with no difference in median OS between sorafenib-pravastatin and sorafenib groups (10.7 months vs. 10.5 months; HR = 1.00; *p* = 0.975) (Jouve et al. (2019))
Pravastatin (40 mg/day), sorafenib, their combination or supportive care	HCC	NCT01357486	Phase II, randomized trial	160	Negative	In the overall Child–Pugh B population, neither sorafenib nor pravastatin seemed to provide benefit. Median OS was similar between the four arms: 3.8 (95% CI: 2.4–6.5), 3.1 (95% CI: 1.9–4.3), 4.0 (95% CI: 3.2–5.5) and 3.5 months (95% CI: 2.2–5.4) in four arms, respectively (Blanc et al. (2021))
Pravastatin (40 mg/day) plus sorafenib	HCC	NCT01418729	Phase II, Randomized, Double-Blind, PC trial	216	No results posted	The purpose of this study was to evaluate the OS in order to assess the efficacy and safety of pravastatin as adjuvant treatment to sorafenib
Atorvastatin (10 mg/day) plus sorafenib	HCC	NCT03275376	Phase II, Randomized	34	No results posted	The aim of this study was to evaluate whether statins improve the tumor responses and overall survival for patients who receive sorafenib therapy for advanced HCC by a prospective randomized controlled study
Atorvastatin (10 mg/day)	HCC	NCT03024684	Phase IV, double-blind, randomized PC trial	Recruiting	No results posted	The aim of this study was to evaluate the effect of atorvastatin for preventing HCC recurrence after curative treatment. The primary endpoint was to compare the 3-year cumulative incidence of recurrent HCC between the intervention group and control counterpart
Pravastatin (40 mg/day) plus sorafenib	HCC	NCT01903694	Phase III, Randomized	474	No results posted	The aim of this study was to evaluate the effect of the combination pravastatin—sorafenib *versus* sorafenib alone on overall survival in patients with hepatocellular carcinoma developing on Child-Pugh A cirrhosis who are unsuitable for curative treatment
Pravastatin plus sorafenib	HCC	NCT01075555	Phase III, Randomized	323	No results posted	The aim of this study was to investigate sorafenib tosylate given together with pravastatin to see how well it works compared with giving sorafenib tosylate alone in treating patients with liver cancer and cirrhosis
Simvastatin (40 mg/day)	High-Risk Compensated Cirrhosis	NCT03654053	Phase III, randomized, double-blind, PC trial	Recruiting	No results posted	The aim of this study was to test whether simvastatin can lower the risk of hepatic decompensation (developing symptoms of cirrhosis) in United States

Abbreviations: HCC, hepatocellular carcinoma; HR, hazard ratio; NR, not reported; OS, overall survival; PC, placebo-controlled; CI, confidence interval; SAM, Sorafenib (S) + Atorvastatin (A) + metformin (M); TAE, transcatheter arterial embolization; TACE, transarterial chemoembolization.

A case-control study using the Taiwan National Health Insurance Research Database has suggested that statins may reduce the risk of liver cancer (Chiu et al., 2011). Later, using the same database, a study has shown that statin use may dose-dependently decrease the risk of HCC in hepatitis B virus (HBV)-infected patients (Tsan et al., 2012); moreover, a similar conclusion with protective effects of statin use was drawn with hepatitis C virus (HCV)-infected patients in this cohort (Tsan et al., 2013). Despite the strong associative indications, further mechanistic evaluation is required. Consistently, statin users with each yearly increment of cumulative defined daily doses (cDDDs) reported a dose-dependent response and reduced HCC risk by 23.6% (Pinyopornpanish et al., 2021). Furthermore, a meta-analysis combining data from 24 studies also demonstrated that statin users showed a 46% decrease in HCC risk, indicative of the potential use of statins as chemoprophylaxis (Islam et al., 2020; Lange et al., 2021). Multiple clinical centers consistently and independently reported the chemo-preventive effects of statins use in HCC among the general population, regardless of the locations of those studies (Björkhem-Bergman et al., 2014; McGlynn et al., 2015; Tran et al., 2020). Statin users have also shown suppressed HCC development in addition to improved liver function and lower cirrhosis risk (Butt et al., 2015). Another study enrolling 1,072 patients with NASH-related advanced liver fibrosis also reported a notable effect of statin in preventing HCC from deterioration (Pinyopornpanish et al., 2021). In summary, the above studies strongly demonstrate the role of statin use in protecting both the general population and HCC risk cohort from HCC occurrence and HCC progression.

Ongoing efforts are driven to further understand the direct effects of statins on the earlier stage of HCC and the prognosis of HCC. The secondary protective effects of simvastatin in cirrhosis are being evaluated in a Phase II clinical trial (NCT02968810). Combined treatment of simvastatin and atorvastatin has decreased the comorbidities for HCC in an Asian cohort (OR = 0.31 and 0.29; 95% CI = 0.14–0.67 and 0.15–0.57, respectively) (Chen et al., 2015b). At present, a multi-center double-blinded randomized trial (Phase IV) has been initiated attempting to determine the potential prevention of atorvastatin for HCC recurrence after curative treatment (SHOT trial; NCT03024684).

#### 3.2.2 Combination of statins with other therapies in HCC

Owing to the comorbidities and other chronic conditions in HCC patients, statins are not used alone but often prescribed together with other frequently or even daily used medications such as aspirin and metformin. A retrospective study of 521 patients demonstrated that the combination use of aspirin and statin is associated with a lower incidence of HCC and this association remained significant in the multivariable model (Singh et al., 2022). Simvastatin, atorvastatin, or rosuvastatin, in combination with metformin, also showed decreased HCC risk among diabetic patients in an Asian cohort; in addition, as for HCC, in particular, only the metformin and simvastatin combination among these combos suggested significantly decreased comorbidities of HCC (Chen et al., 2015b). NAFLD is known to develop liver inflammation and progress to NASH, fibrosis, cirrhosis or HCC. For the treatment of NAFLD/NASH at high risk of CVD or HCC, statins alone or together with anti-diabetic PPAR-gamma agonist pioglitazone and other drugs, were primarily recommended for the primary or secondary prevention of CVD, in addition to cirrhosis avoidance, liver transplantation, and HCC, according to a statement from official guidelines (Athyros et al., 2017).

For intermediate-stage HCC, transarterial chemoembolization (TACE) is the most common bridging therapy as a standard local-regional treatment before liver transplantation. Compared to chemoembolization alone, combination therapy of chemoembolization and pravastatin greatly improves survival of advanced HCC (Graf et al., 2008). During the 5-year observation of HCC patients, 23.7% of patients treated by TACE alone and 36.5% of patients treated by TACE plus pravastatin survived, and median survival was significantly longer in HCC patients treated by the combination than in patients treated by TACE alone (Graf et al., 2008).

Currently, a body of clinical trials addressing the interventional impacts of statins alone or with non-immunotherapy treatment in HCC have been initiated (Table 2). So far, 6 of the 11 trials have not reported any results, and one trial has indicated benefits of atorvastatin in reducing sorafenib-related side effects. In the rest 4 trials, all trials used pravastatin as treatment for advanced HCC, and pravastatin plus conventional transcatheter arterial embolization (TAE) (plus 5-FU) or TACE in 2 of the trials have observed prolonged survival of patients with advanced HCC (Kawata et al., 2001; Graf et al., 2008); however, the other 2 trials concluded more recently (2019 and 2021) using pravastatin in combination with sorafenib showed no benefits in OS, and even the protective effects of sorafenib seem to disappear or be very subtle (Jouve et al., 2019; Blanc et al., 2021), which is difficult to interpret as a whole. Treatment strategies among these trials differ, and the sample size for conclusion and the subpopulation at various disease stages or with varying liver function, even though all patients at advanced HCC, could contribute to the unexpected ineffectiveness. More results from the rest ongoing trials are being anticipated.

### 3.3 Adverse effects of statins in HCC

Statin-associated cardiovascular benefits, including declined risks of major coronary events and revascularization as well as the risk of stroke, far outweigh the potential risks. However, after statins have been prescribed for clinical use for several decades, statin is shown to be not all good. Statin-associated muscle symptoms (SAMS) are the most common toxicity of statins, as shown by manifestations of myalgia, myopathy, myositis with increased creatine kinase (CK), or rhabdomyolysis (Ward et al., 2019), and SAMS risk appears to link with systemic exposure to higher doses of statins (Armitage et al., 2010). As reported in a randomized controlled trial, two patients with advanced liver disease, out of 69 patients in the simvastatin group, receiving simvastatin 40 mg/day experienced rhabdomyolysis, with no such sign found in the placebo group (Abraldes et al., 2016).

Although the statins-induced liver injury is relatively uncommon (<1.2/100,000 users) and likely idiosyncratic in nature (Björnsson et al., 2012), the risk of hepatotoxicity has been reported from time to time in statin users, therefore physicians should be cautious when prescribing statins to patients with liver diseases (Rzouq et al., 2010; Blais et al., 2016). An earlier assessment indicates that decompensated cirrhosis or acute liver failure, rather than chronic liver disease or compensated cirrhosis, are contraindications for statin use (Bays et al., 2014). Mechanistically, individuals with advanced cirrhosis are challenged with elevated drug exposure resulting from delayed statin clearance, deficiency of cytochrome P450 3A4 which is responsible for drug metabolism in the liver, and disrupted transporter activity (Pose et al., 2019), therefore facing a higher risk of SAMS.

Despite the harmful impacts on muscle and liver with statin therapy, concerns have emerged regarding statin-related risk of new-onset diabetes mellitus, cognitive impairment, and hemorrhagic stroke, as well as the risk of extremely low levels of LDL cholesterol (LDL-C) (Adhyaru and Jacobson, 2018). For instance, the incidence of new-onset diabetes mellitus is approximately 0.1% per year and 0.2% per year with moderate-intensity and high-intensity statin therapy, respectively. Consequently, balancing the clinical benefits and potential risks, statins should be provided diligently and wisely to patients to ensure that they adhere to therapy regimens (Adhyaru and Jacobson, 2018).

## 4 TIME and targeted therapies in HCC

### 4.1 The TIME of HCC

When the liver lesions develop from liver cirrhosis to HCC, numerous immune cells progress to dysfunction, in a manner of being either abnormally inactivated or overactivated. As a result, TIME is formed as a key component of TME, bridging the interplay between tumor cells and immune cells, which is crucial for HCC development and somehow dictates the immunotherapy outcomes. Characterizing the immunological networks present in the TIME of HCC will facilitate the understanding of liver immunity and the principal mechanisms of both spontaneous and therapy-induced immune responses.

The activation of dendritic cells (DCs) contributes to the activation of CD8^+^ T-cells, while regulatory B-cells (Bregs) inactivate CD8^+^ T-cells (Kotsari et al., 2023). In HCC, decreased antigen presentation is found in DCs (Hilligan and Ronchese, 2020; Kotsari et al., 2023). In primary HCC patients, TAM density predicts poor prognosis related to vascular invasion, tumor multiplicity, and fibrous capsule formation (Oura et al., 2021). Pro-inflammatory cytokines induced by toll-like receptor (TLR) ligand and Th1 response index, such as IFN-α/β, and IFN-γ, could activate M1 macrophages to differentiate into M2 macrophages (Oura et al., 2021), which enhances the recruitment and growth of Tregs, resulting in an aggressive phenotype, poor OS, and rapid recurrence (Dong et al., 2016), by downregulating CD8^+^ T cells, DCs, and NK cells HCC (Langhans et al., 2019). Tumor-associated neutrophils (TANs) produced chemokines such as CCL2 and CCL17, which later recruited TAMs and Tregs and promoted tumor growth in HCC (Zhou et al., 2016). CAFs could produce inflammatory cytokines, growth factors, and chemokines to promote HCC development and metastasis (Foerster et al., 2022; Xu et al., 2022); moreover, CAFs activate TANs and promote the differentiation of monocytes into MDSCs (Kotsari et al., 2023). The number of B-cells in HBV-positive HCC correlates with smaller tumor size, compromised vascular invasion, and augmented infiltration of CD8^+^ T lymphocytes, furthermore, an increased population of B-cell subsets notably prolonged HCC patients’ survival (Zhang et al., 2019; Kotsari et al., 2023), suggestive of protective benefits of B cell in HCC. These above-described immunocomponents, alongside the tumor cells, and a variety of inflammatory molecules compose a complex microenvironment for immune response and regulation, advances of which have greatly facilitated the immunoregulation directed therapies, certainly not limited to HCC. In recent years, immunotherapies in combination with conventional treatments such as anti-angiogenic drugs in HCC have achieved extraordinary success, although the effects of immunotherapy alone as treatment have been of relatively infrequent benefit. Here we only briefly introduce the most popular and well-accepted immunotherapies [reviewed elsewhere (Oura et al., 2021; Li et al., 2023a)] in HCC.

### 4.2 Immunotherapies in HCC

After tumors escape from immune control, immunotherapies pioneered by ICIs and related applications are aiming to activate the immune system to recognize, target, and eliminate cancer cells. In addition to ICIs, other immunotherapies such as ACT and cancer vaccines also demonstrate promising efficacy in HCC.

#### 4.2.1 Immune checkpoint inhibitor (ICI) therapies in HCC

In recent years, ICI therapies have been well recognized as a main component in systemic first-line treatment of HCC due to considerably less systemic side effects and more durable responses compared with other conventional therapies (He and Xu, 2020). Programmed cell death protein-1 (PD-1), programmed death ligand-1 (PD-L1) and cytotoxic T lymphocyte-associated protein 4 (CTLA-4) signaling represent the most prominent and well-studied immune checkpoints. By inhibiting these immunoreceptors, ICIs boost the antitumor activities of host immune cells to prevent the metastasis of cancer cells (He and Xu, 2020). PD-1 on the surface of immune cells binds to PD-L1 in tumor cells, leading to tumor immune evasion (Chen et al., 2023b). Blocking the PD1/PD-L1 interaction so far appears to be one of the most effective immunologic treatments for cancers (Llovet et al., 2022; Zhang et al., 2023). The immune checkpoint regulator CTLA-4 is exclusively expressed in T cells and impedes the effector functions of these T cells (Bulaon et al., 2023). Although the response to immunotherapy in HCC is limited due to several reasons, the safety and efficacy of these ICI-based therapies have been widely confirmed in HCC-relevant trials (Borghaei et al., 2015; Ribas and Wolchok, 2018; Li et al., 2023a). Major inhibitors relating these immunoreceptors, in the format of monoclonal antibodies, include PD-1 inhibitors nivolumab, pembrolizumab and sintilimab, PD-L1 inhibitor atezolizumab and durvalumab, and CTLA-4 blocker tremelimumab and ipilimumab, etc. (Li et al., 2023a; Shannon et al., 2023).

The effects of ICI monotherapy were first investigated in HCC and have been approved by the FDA. The efficacy of anti-PD-1 monoclonal antibody nivolumab in advanced HCC has been assessed in a phase I/II study, which showed an objective response rate (ORR) of 20%, a disease control rate (DCR) of 64% and a median OS of 13.2 months (El-Khoueiry et al., 2017). Another phase II trial showed that in patients with advanced HCC who had been previously treated with a multi-target kinase inhibitor sorafenib for HCC, PD-1 inhibitors pembrolizumab suggested an ORR rate of 17% and 77% of patients demonstrated sustained response for more than 9 months (Zhu et al., 2018).

Conventional treatments such as VEGF inhibitors and multi-kinase inhibitors are usually offered as a foundational treatment when the efficacy of additional ICI therapies is evaluated. A phase II/III study assessed the efficacy of PD-1 inhibitor sintilimab plus IBI305, a bevacizumab (VEGF monoclonal antibody) biosimilar, *versus* sorafenib, as a first-line therapy for unresectable HBV-associated HCC. As a result, patients in the sintilimab and IBI305 combination group indicated a significantly longer median PFS (4.6 months) than sorafenib group patients (2.8 months) (Rossi et al., 2021). Similarly, the IMbrave 150 clinical trial demonstrated that PD-L1 inhibitor atezolizumab combined with bevacizumab had improved PFS and OS *versus* sorafenib treatment in HCC patients (Finn et al., 2020; Roy, 2022). It is worth noting that these updated results from this key study confirm the combination as the first-line standard of care for advanced HCC (Finn et al., 2020; Roy, 2022).

Combined ICI immunotherapies have also been widely explored in HCC. PD-1 inhibitor nivolumab monotherapy has been provento improve prognosis in HCC, and the addition of CTLA-4 inhibitor ipilimumab seemed to augment the impact, suggested by elevated ORR and OS in the combination group (Yau et al., 2022). In line with that, another CheckMate 040 clinical trial also suggested nivolumab plus ipilimumab had manageable safety, promising ORS, and durable responses (Yau et al., 2020). A phase III HIMALAYA clinical trial has also shown that combination therapy of PD-L1 inhibitor durvalumab and CTLA-4 blocker tremlimumab correlates with improved ORR and OS compared with sorafenib treatment (Abou-Alfa et al., 2022).

#### 4.2.2 Non-ICI therapies

Chimeric antigen receptor (CAR)-T cells and T cell receptor (TCR) engineered T cells are 2 cell types of ACT therapy that are applied in the therapy of HCC. Currently, about 24 clinical trials related to CAR-T cell therapy for HCC are in phase I/II (Gao and Zuo, 2023). Among these, glypican-3 (GPC3) is the main target. In addition, other targets include alpha-fetoprotein (AFP), NK group 2, member D ligand, mucin 1 glycoprotein 1, claudin18.2, CD147, CD133, etc. Efficient multi-epitope peptide vaccines against HCC have started to be designed. As the main target of adoptive T cell therapy for HCC, GPC3, and AFP have been employed in designing HCC vaccines. A phase I trial in advanced HCC has demonstrated that GPC3 peptide vaccine-induced GPC3-specific CTLs that could infiltrate into the HCC tissues, leading to improved OS induced by GPC3-based vaccine in advanced HCC (Tsuchiya et al., 2017). Besides, an AFP-based vaccine has been designed for the treatment of AFP-positive HCC (Lu et al., 2023). Recently, most of cancer vaccines were generated against HCC, such as the VEGF vaccine, the DC-based nano-vaccine, as well as a combination of vaccines and ICIs, and all applications have demonstrated the capacity to halt HCC progression (Gao and Zuo, 2023; Lu et al., 2023). Virotherapy presents a novel immunotherapy modality for HCC. Oncolytic virotherapy (OVT) effectively induces antitumor responses through selective replication of oncolytic virus in cancerous tissues and killing HCC cells (Li et al., 2023b).

## 5 Interaction of statin treatment and immunotherapy in HCC

### 5.1 Crosstalk of TIME and lipid metabolism in HCC

**FIGURE 3:**
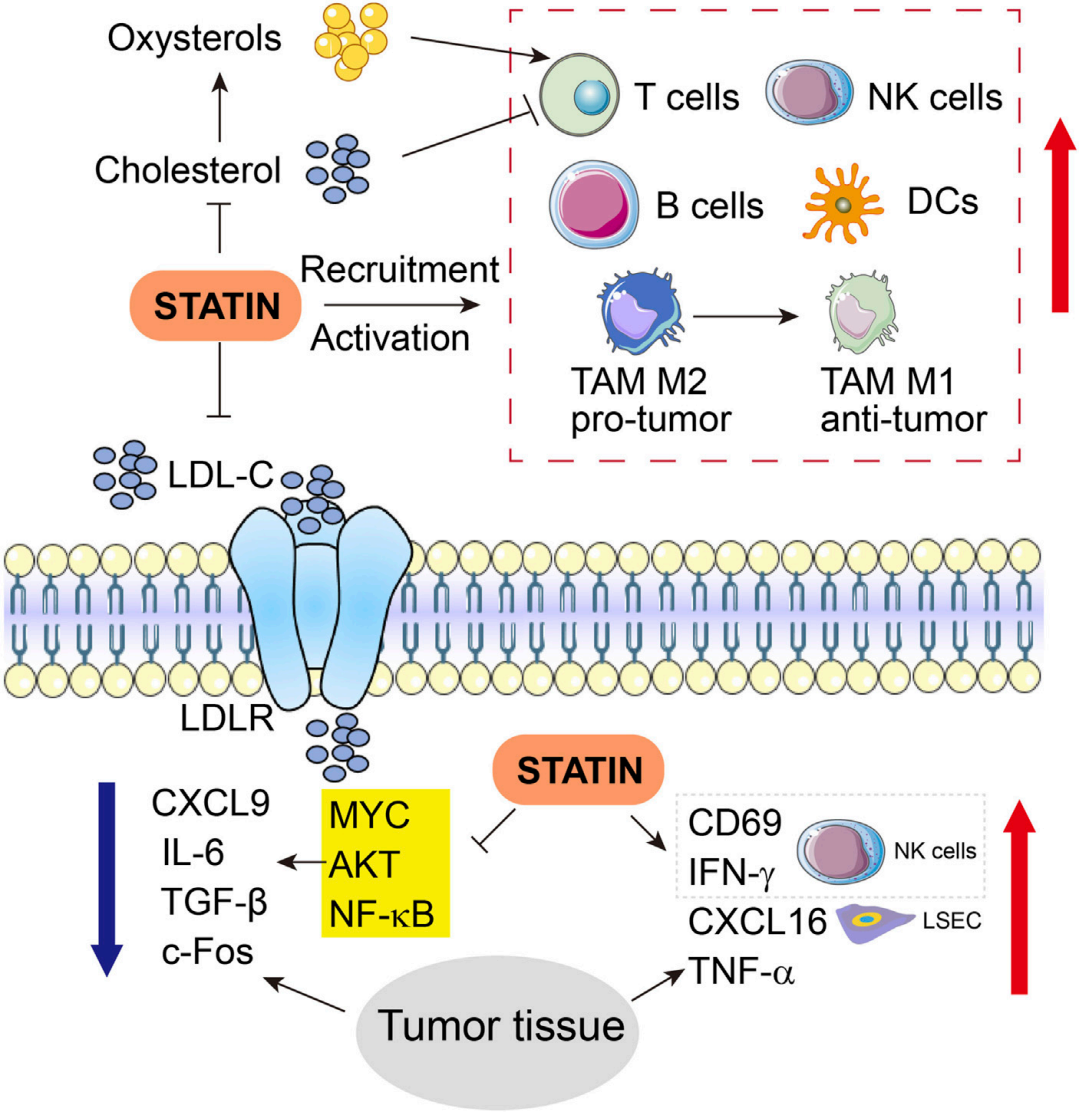
Engagement of immune cells and related molecules and cytokines in anti-tumor functions of statins in HCC. Upward red arrows indicate activation or increased expression, whereas downward blue arrow indicates inhibition or suppressed expression. LSEC, liver sinusoidal endothelial cells.

In TIME, molecules related to lipid metabolism and their metabolites may directly or indirectly impact the state of tumor immune responses. Low expression of transmembrane protein coiled-coil domain containing 25 (CCDC25) on HCC cells leads to metabolic disorders such as dysregulation of FA, and CCDC25is shown to affect the sensitivity of HCC to targeted therapy, infiltration of immune cells as well as expression of immune checkpoints. Moreover, CCDC25 abundance positively correlates with the infiltration of CD8^+^ T cells, macrophages, and DCs, but is negatively associated with infiltration of Tregs and expression levels of immune checkpoints such as CTLA4 (Dickson, 2020; Yang et al., 2020; Deng et al., 2022). Blockade of immune escape of tumors also involves CCDC25 via recruiting more tumor killer cells, inactivating immunosuppressive cells, and direct inhibition of immune checkpoints (Dickson, 2020; Oura et al., 2021; Deng et al., 2022; Liang et al., 2023a). As a significant component of membrane lipids, cholesterol plays a vital part in the formation of immune synapses of T cells and thereby regulates the functions of the T cell receptor (Molnár et al., 2012). In contrast, the upregulated production of oxysterols that are oxidized from cholesterols in turn inhibits the T cell functions through the liver X receptor signaling (Figure 3) (Huang et al., 2020).

### 5.2 Effects of statins on immune microenvironment in HCC

Apart from the above-mentioned anti-tumor effects of statins in HCC involving inflammation and non-inflammation pathways, the impacts of statins on the immune microenvironment would guide the delicate interplays between immune cells and tumor cells; furthermore, this knowledge may also provide mechanistic insights and clues if statins could favor or boost the development of future immunotherapy in HCC.

Simvastatin has been shown to indirectly regulate the recruitment and activation of dozens of relevant immune cells (Figure 3). Transcript expression and protein expression of chemokine CXCL16 are augmented by simvastatin in liver sinusoidal endothelial cells (LSEC) SK-Hep1 in a dose-dependent manner. However, although simvastatin alone fails to alter the activity of natural killer T (NKT) cells, CXCL16 overexpression induced by simvastatin recruits and potentiates NKT cells to the liver, and later simvastatin upregulates CD69 and IFN-γ expression in the NKT cells when SK-Hep1 and NKT cells are co-cultured, and thoroughly activates NKT cells (Yu et al., 2022). Further evidence shows that simvastatin treatment increased the production of the immunostimulatory cytokines such as CXCL16, IFN-γ, and TNF-α, while suppressing the expression of immunosuppressive cytokines including CXCL9, IL-6, and TGF-β in HCC tumor tissues (Yu et al., 2022). Pitavastatin treatment can change the cytokine microenvironment by inhibiting the cytokine production in HCC, partially due to NF-κB activation and subsequent downstream IL-6 expression triggered by TNF-α in HCC cells (Figure 3) (Wang et al., 2006).

Fos-dependent inflammation contributes to HCC progression. Knockout of c-Fos in hepatocytes protects the liver against HCC initiation, while overexpression of c-Fos in the liver accelerates the malignant transformation of HCC, as manifested by necrotic foci, accumulated CD45^+^ cells, reduced NK and B cells, increased circulating leukocytes, infiltration of immune cells and accumulated lesions in hepatocytes. In this manner, c-Fos-dependent HCC progression is blocked by statin treatment, which has also modulated the components of the immune system in the TIME of HCC (Figure 3) (Bakiri et al., 2017).

### 5.3 Combinational effects of statins and immunotherapies in non-HCC cancers

Dyslipidemia often takes place either before the onset of cancer in chronic condition or as a concomitant outcome as metabolic dysfunction after cancer initiation, therefore, cholesterol-lowering statins are a regularly prescribed medication that has to be continuously used (Lamon-Fava, 2013; Adhyaru and Jacobson, 2018; Ward et al., 2019). In this respect, the association of statin treatment with available immunotherapies in cancers such as lung cancer, breast cancer, advanced renal cell carcinoma, head and neck cancer, etc., has been broadly investigated.

Takada et al. (2022) examined 390 patients with advanced or recurrent non-small-cell lung cancer (NSCLC) who were treated with anti-PD-1 therapy in clinical practice and found that patients receiving anti-PD-1 therapy combined with statin treatment have much longer OS than those without statin treatment. However, another study showed that when the two groups of PD-L1 treatment with or without statin were compared, median PFS was 17.57 months and 9.57 months in the statin group and non-statin group, respectively (*p* < 0.001); median OS was significantly (*p* < 0.001) higher in the statin group than the non-statin group 19.94 and 10.94 months, respectively (Rossi et al., 2021). Although further prospective randomized trials are required, these strong associations together confirm that ICI treatment combined with statins in NSCLC patients may remarkably improve survival and prognosis, suggesting that the antitumor functions of statins synergize the benefits of ICI therapy in prevalent lung cancer.

In a cohort of metastatic renal cell carcinoma receiving PD-1 inhibitor nivolumab, 27% were statin users and 73% were non-statin users. The median OS and PFS were longer in the statin user group than in the non-statin users. Interestingly, in both patients aged ≥70 years and <70 years, the longer median OS and PFS were associated with longer statin exposure (Santoni et al., 2022). In conclusion, overall clinical benefits were greater in the statin user group than non-statin user group (71% and 54%), again strongly supporting the benefit of statin in PD-1-directed immunotherapy (Santoni et al., 2022).

Furthermore, in breast cancer, atorvastatin promotes cytotoxic T-cell activity, inhibits the immune evasion of T cells, and enhances antitumor immune response, thereby boosting the efficacy of anti-PD-L1 therapy (Choe et al., 2022). In addition, daily oral simvastatin or lovastatin combined with PD-1 blockade in mice promoted tumor control and extended survival, notably, lovastatin plus anti-PD-1 treatment leads to rejection of oral cancer tumors of the head and neck cancer in 30% of mice. The underlying protective effects are likely owing to that combination therapy enhances T cell activation and promotes predominant shifts of macrophage from M2 to M1 status, therefore, exerting resistance against head and neck cancer (Kansal et al., 2023) (Figure 3). In summary, these evidences together imply a consistent advantage of statin use in improving the therapeutic response of immunotherapy in multiple cancer types, via interacting with a range of immune cells, although detailed mechanisms are still lacking.

### 5.4 Combination of systemic therapy and immunotherapy in HCC

Before ICI immunotherapy had modified the management of HCC in the past few years, around 50% of patients with HCC received systemic therapies, generally, sorafenib or lenvatinib in the first line, followed by regorafenib, cabozantinib or ramucirumab as the second. Combinational regimens have been widely shown to yield significantly improved OS and superior PFS, thereby receiving rapid FDA approval (Llovet et al., 2022). For instance, generally used tyrosine kinase inhibitors (TKIs) for systemic treatment including sorafenib and lenvatinib, in combination with ICIs including nivolumab and pembrolizumab have been approved for the treatment of advanced HCC because of their noteworthy antitumor efficacy (Lee et al., 2022). Advances and benefits of immunotherapies along with other conventional treatment strategies have been well discussed in a broad therapeutic view in cancers including but not limited to HCC (Llovet et al., 2022).

Typical TKI, sorafenib, combined with PD-1-based ICIs for advanced HCC has been proven to be safe and effective, as the median PFS of combination treatment was greatly longer than the PD-1 monotherapy, The median OS of the combination treatment group (21.63 months) was also longer than the PD-1 group (16.43 months) (Qin et al., 2022). Another TKI Lenvatinib, also an angiogenesis inhibitor, in combination with ICI therapy has demonstrated a synergistic antitumor effect, as VEGFA inhibition promotes the infiltration and survival of CTLs, and the meantime mitigates recruitment of Treg lymphocytes, leading to the more advantageous immune microenvironment for antitumor activity of ICI therapy (Hilmi et al., 2019). Lenvatinib was also found to be superior to sorafenib as the first-line treatment of HCC in regard to OS improvement (Kudo et al., 2018). Lenvatinib and anti-PD-1 antibody together robustly suppressed tumor growth, induced vascular normalization, and improved anti-PD-1 therapeutic efficacy in HCC (Yang et al., 2023). In a cohort of 139 male Chinese patients with advanced HCC, the median OS in the combined treatment group (PD-1 inhibitor sintilimab plus Lenvatinib) and Lenvatinib monotherapy group were 21.7 months and 12.8 months, and the median PFS were 11.3 months and 6.6 months, respectively. This combination regimen has shown acceptable efficacy and safety in practice and obviously improved long-term outcomes than monotherapy with either Lenvatinib or PD-1inhibitor (Zhao et al., 2022). Novel TKI regorafenib also augments the effects of ICI therapy against HCC (Xie et al., 2023). Afatinib, a second-generation EGFR-TKI, exhibits considerable inhibitory impacts on liver cancer cells and enhances the PD-L1 presentation in tumor cells. Afatinib combined with anti-PD1 treatment also notably enhances the immunotherapeutic effect in HCC (Yu et al., 2023).

Besides TKI-based therapies for HCC, there are many preclinical studies showing other non-TKI compounds are also potential candidates for adjuncts of immunotherapy in HCC. In particular, many of these drugs, similar to statins, are regularly seen and prescribed for chronic metabolic syndromes. The effect of metformin plus anti-PD-1 is enhanced than anti-PD-1 therapy against liver tumors in NASH-HCC murine models (Wabitsch et al., 2022). Aspirin enhanced the anti-PD-L1 immunotherapeutic efficacy, and combination therapy significantly induced HCC tumor regression and extended the lifespan of tumor-bearing mice (Lin et al., 2023). Moreover, abrine, a specific inhibitor of indoleamine-2,3-dioxygenase 1 (IDO1) and also a major player in immunosuppression in tumors, exerts profound liver-protective functions in immunotherapy. The combination of abrine and anti-PD-1 antibody treatment synergistically repressed the tumor growth in HCC by inducing CD4^+^ or CD8^+^ T cells, decreasing Foxp3^+^ Treg cells, and inhibiting immune-suppressive molecules such as IDO1, CD47, and PD-L1(Liang et al., 2023b). Taken together, notable successes have been obtained in clinical trials when HCC is treated with immunotherapies combined with systemic monotherapy that has been used alone in the past.

### 5.5 Effects of statins on immunotherapies in HCC

Recently, although statins have already been applied in other non-HCC cancers as adjuncts of immunotherapy, and obvious benefits have been attained, such applications in the clinical treatment of HCC are still lacking. Preclinical studies in HCC mice revealed that either simvastatin or PD-L1 antibody alone indicated a slight but significant influence in tumor suppression (*p* < 0.01), whereas the combination of simvastatin and PD-L1 antibody significantly inhibited HCC tumor progression (*p* < 0.001), and the OS in the combination therapy were prolonged almost 2 times compared with the non-drug control (*p* < 0.001) (Yu et al., 2022). Furthermore, simvastatin treatment combined with PD-L1 antibody could not only improve the prognosis of the intrahepatic inoculation HCC model but also achieve satisfactory therapeutic efficacy in model of advanced HCC (Yu et al., 2022). Inspired by other combinations of statin use with immunotherapy in other cancer types, data from this study, although from a preliminary study, has underlined the great potential of statins as adjuncts for immunotherapy in HCC.

Furthermore, a prospective observational trial designed to evaluate the safety and efficacy of ICI therapy in combination with statins in treating NSCLC is now recruiting patients. We anticipate that preclinical and clinical explorations of statin/ICI therapy regimens for both HCC and non-HCC cancers will soon, embrace unprecedented new opportunities, certainly facing concomitant challenges ahead.

In addition to statins, other lipid-lowering drugs, such as PCSK9 inhibitors, fibrates, and ezetimide, may also impact or potentiate ICI therapy. In particular, immunomodulatory effects of PCSK9 inhibition are being studied shortly in cardiovascular disease and inflammation-related conditions, in addition to a phase II trial in NSCLC aiming to evaluate the anti-tumor activity of the combination of anti-PCSK9 and anti-PD-1 antibody therapy.

## 6 Conclusion and future perspectives

In the past decades, statins, traditionally used because of their cholesterol-lowering properties, have demonstrated multifaceted effects on the immune system in general cellular homeostasis, including modulation of T cell responses and anti-inflammatory properties. The exploration of statins as potential adjuncts in immunotherapies for HCC and other cancer types has yielded promising results. By enhancing immune responses via cytokine stimulation, statins can enhance the efficacy of immunotherapies such as ICIs in HCC. Such a combination approach, by incorporating statins into the existing immunotherapeutic regimens, may the hold key to improving treatment outcomes in HCC. However, it’s crucial to acknowledge that more extensive preclinical investigations are prerequisites to provide more mechanistic basis for future clinical trials, before adding statins into immunotherapy regimens in HCC.

Such an integration strategy for HCC represents an exciting avenue from several future perspectives. First, future trials should assess the safety and efficacy of combining statins with various immunotherapeutic agents, with a focus on patient stratification to identify those who might benefit most. In addition, mechanistic studies should delve deeper into the immunomodulatory effects of statins in HCC, to resolve the precise pathways and cellular interactions that contribute to enhanced outcomes of immunotherapies. Furthermore, the development of novel statin derivatives with improved bioavailability and reduced side effects could broaden the feasibility of combination therapy. Lastly, predictive biomarkers to identify HCC patients who are most likely to respond favorably to statin-immunotherapy combinations could be a priority to investigate. In conclusion, the future of statins in immunotherapies for HCC holds great promise, likely to refine the treatment landscape for this challenging malignancy and improve the treatment outcomes in patients.
